# Supplementary material for: Spermidine suppresses DC activation via eIF5A hypusination and metabolic adaptation
Source: Discov Immunol. 2025 May 15;4(1):kyaf009. doi: 10.1093/discim/kyaf009 (PMC12159527; doi:10.1093/discim/kyaf009)
Supplement: kyaf009_suppl_Supplementary_Figure_S1 [file kyaf009_suppl_supplementary_figure_s1.pdf]

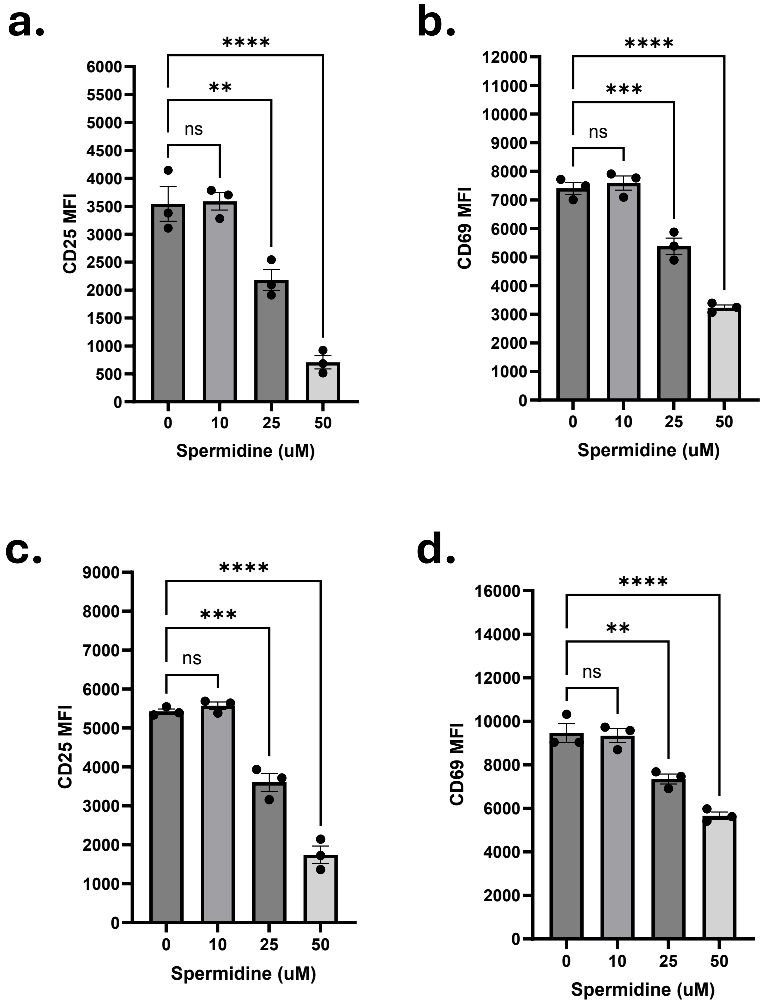


**Supplementary Figure 1: Spermidine inhibits BMDC driven activation of CD4 T cells.** T cell (OTII) and dendritic cell (BMDC) co-cultures were incubated with various concentrations (10 µM – 50 µM) of spermidine and activated with 2 µg/mL OVA peptide (a, b) or 2 µg/mL mitogen Con A (c, d) for 24 hours. T cell activation was determined by analysis of CD25 (a, c) and CD69 (b, d) expression, expressed as mean fluorescence intensity. n = 3, representative of two independent experiments. One Way ANOVA; * < 0.05; ***<0.001; ****<0.0001.
