# Supplementary material for: Spermidine suppresses DC activation via eIF5A hypusination and metabolic adaptation
Source: Discov Immunol. 2025 May 15;4(1):kyaf009. doi: 10.1093/discim/kyaf009 (PMC12159527; doi:10.1093/discim/kyaf009)
Supplement: kyaf009_suppl_Supplementary_Figure_S2 [file kyaf009_suppl_supplementary_figure_s2.pdf]

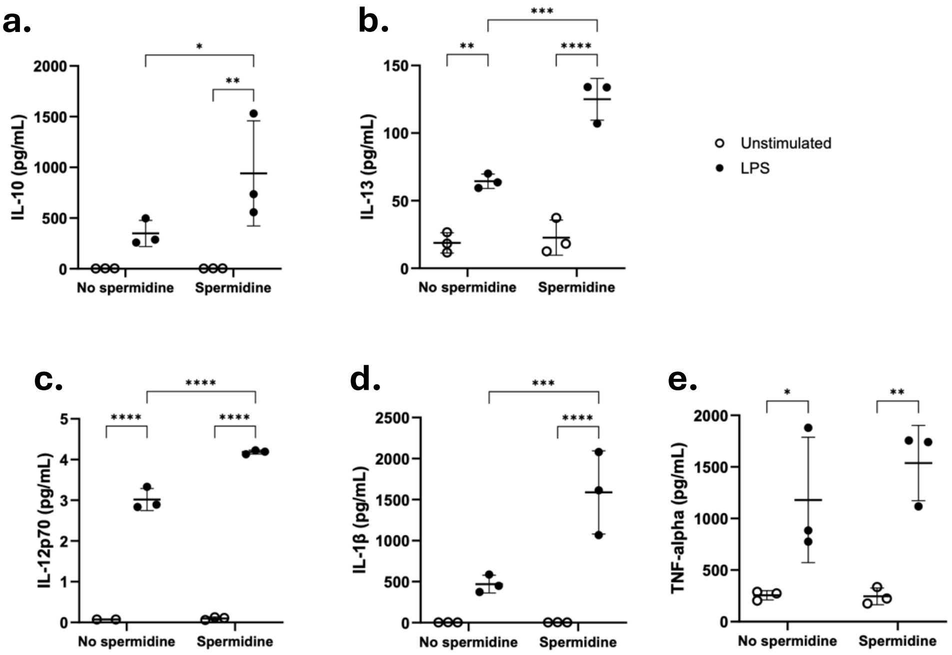

Supplementary Figure 2: Spermidine enhances pro- and anti-inflammatory cytokine production by BMDC. Bone marrow derived dendritic cells (BMDCs) were incubated with or without 0.1mM spermidine in the presence or absence of LPS for 24 hours. IL-10 (a), IL-13 (b), IL-12 (c), IL-1b (d) and TNF (e) levels were measured in supernatants using a Luminex assay. n=3. One Way ANOVA, *<0.05, **<0.01, ***<0.001, ****<0.0001
