# Supplementary material for: Spermidine suppresses DC activation via eIF5A hypusination and metabolic adaptation
Source: Discov Immunol. 2025 May 15;4(1):kyaf009. doi: 10.1093/discim/kyaf009 (PMC12159527; doi:10.1093/discim/kyaf009)
Supplement: kyaf009_suppl_Supplementary_Figure_S3 [file kyaf009_suppl_supplementary_figure_s3.pdf]

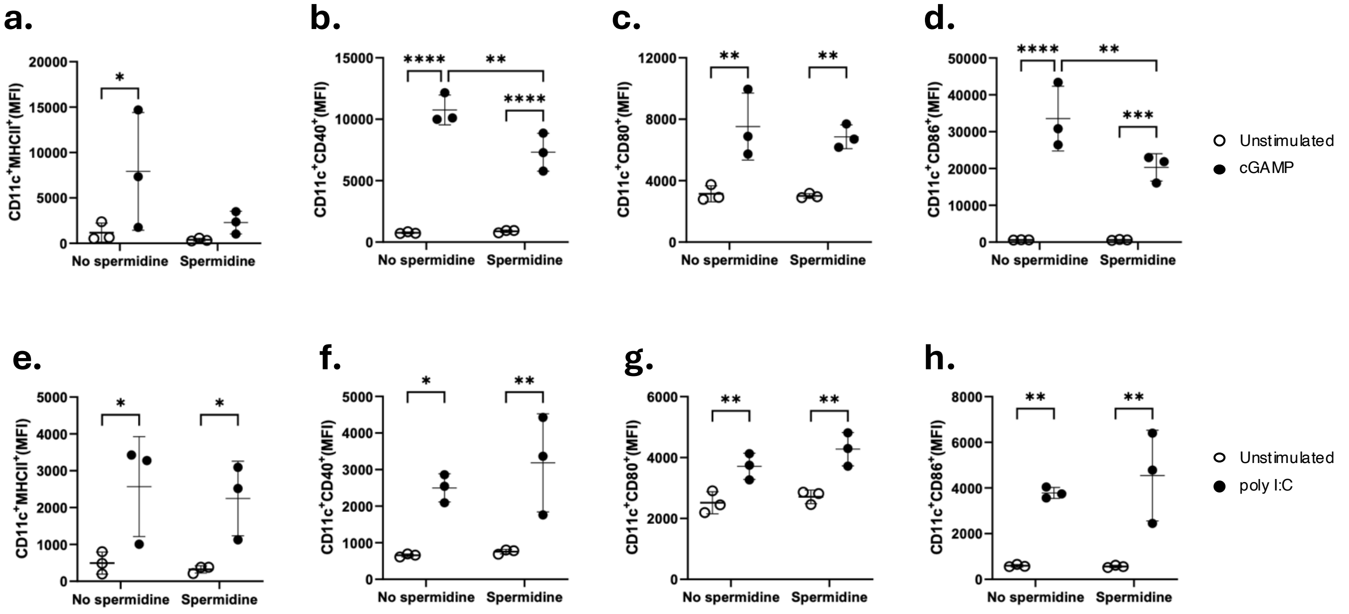

Supplementary Figure 3: Spermidine inhibits activation of CD11c+ bone marrow DCs in response to cGAMP, though not poly I:C. BMDC were incubated with or without 0.1mM spermidine in the presence of 50 µg/ml cGAMP (a-d) or 10 µg/ml polyI:C (e-f) for 24 hours. The BMDCs were then harvested, stained and analysed by flow cytometry. The median fluorescent intensities (MFI) of (a, e) MHCII, (b, f) CD40, (c, g) CD80 and (d, h) CD86 were assessed between the treatment groups. n=3. One Way ANOVA, ns = no significance, *<0.05, **<0.01, ***<0.001, ****<0.0001
