# Supplementary material for: Spermidine suppresses DC activation via eIF5A hypusination and metabolic adaptation
Source: Discov Immunol. 2025 May 15;4(1):kyaf009. doi: 10.1093/discim/kyaf009 (PMC12159527; doi:10.1093/discim/kyaf009)
Supplement: kyaf009_suppl_Supplementary_Figure_S4 [file kyaf009_suppl_supplementary_figure_s4.pdf]

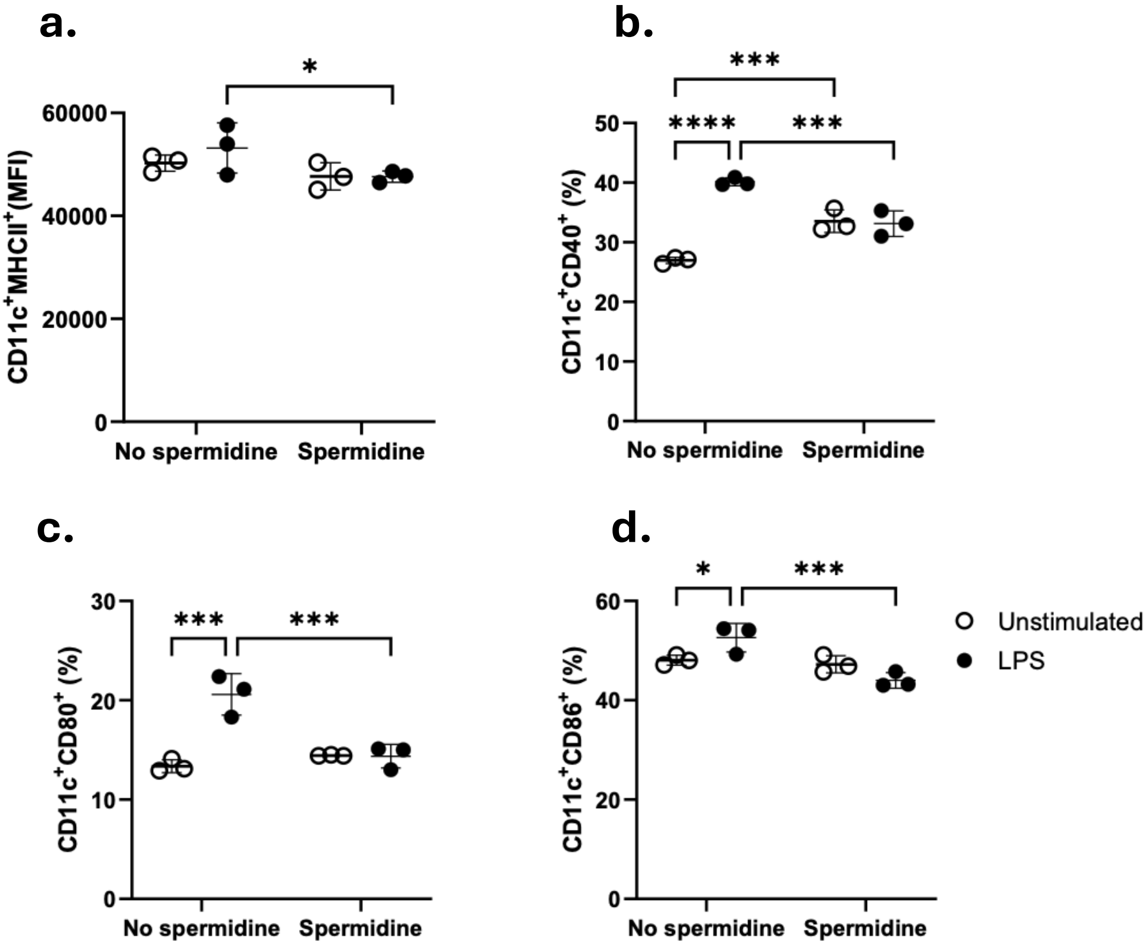

Supplementary Figure 4: Spermidine inhibits activation of CD11c+ splenic DCs in response to LPS. CD11c+ splenic DC were isolated from mice treated with Flt3L for 10 days (10µg of Flt3L/day/mouse via ip). DC were incubated with or without 10 µM spermidine in the presence of 20ng/ml LPS or media only for 24 hours. The DCs were then harvested, stained and analysed by flow cytometry. The percentage of cells expressing (a) MHCII, (b) CD40, (c) CD80 and (d) CD86 were compared between the different treatment groups (n=3). One Way ANOVA, *<0.05, **<0.01, ***<0.001, ****<0.0001.
